# QuEChERS Purification Combined with Ultrahigh-Performance Liquid Chromatography Tandem Mass Spectrometry for Simultaneous Quantification of 25 Mycotoxins in Cereals

**DOI:** 10.3390/toxins8120375

**Published:** 2016-12-15

**Authors:** Juan Sun, Weixi Li, Yan Zhang, Xuexu Hu, Li Wu, Bujun Wang

**Affiliations:** 1 Institute of Crop Sciences, Chinese Academy of Agricultural Sciences, Beijing 100081, China; gwzx848@163.com (J.S.); liweixi@caas.cn (W.L.); zhangyan06@caas.cn (Y.Z.); huxuexu@caas.cn (X.H.); wuli5151@126.com (L.W.); 2 Laboratory of Quality and Safety Risk Assessment for Cereal Products (Beijing), Ministry of Agriculture, Beijing 100081, China

**Keywords:** ultrahigh performance liquid chromatography tandem mass spectrometry (UPLC–MS/MS), QuEChERS, mycotoxin, cereals

## Abstract

A method based on the QuEChERS (quick, easy, cheap, effective, rugged, and safe) purification combined with ultrahigh performance liquid chromatography tandem mass spectrometry (UPLC–MS/MS), was optimized for the simultaneous quantification of 25 mycotoxins in cereals. Samples were extracted with a solution containing 80% acetonitrile and 0.1% formic acid, and purified with QuEChERS before being separated by a C18 column. The mass spectrometry was conducted by using positive electrospray ionization (ESI+) and multiple reaction monitoring (MRM) models. The method gave good linear relations with regression coefficients ranging from 0.9950 to 0.9999. The detection limits ranged from 0.03 to 15.0 µg·kg^−1^, and the average recovery at three different concentrations ranged from 60.2% to 115.8%, with relative standard deviations (RSD%) varying from 0.7% to 19.6% for the 25 mycotoxins. The method is simple, rapid, accurate, and an improvement compared with the existing methods published so far.

## 1. Introduction

Mycotoxins can be analyzed by various methods, including thin layer chromatography (TLC) [1], enzyme-linked immunosorbent assay [2], gas chromatography [3,4], and immunoaffinity column/high-performance liquid chromatography with fluorescence and diode array detection [5,6]. The specific determination of multiclass mycotoxins in cereals requires highly selective and sensitive techniques such as ultrahigh-performance liquid chromatography tandem mass spectrometry (UPLC–MS/MS) [7,8,9,10,11] and direct analysis in real time (DART) ionization coupled to an (ultra)high-resolution mass spectrometer based on orbitrap technology (orbitrap MS) [12].

Several methods have been developed to purify mycotoxins from crude samples such as solid-phase extraction (SPE) [13], immunoaffinity columns (IACs) [14], and MycoSep columns [15]. A direct and simple method for the purification of multiple mycotoxins is challenging because of their diverse chemical structures and properties. The SPE cartridge is the most commonly used purification column, but the purification is tedious and time consuming. The most prominent feature of IACs is their low matrix effects, high selectivity, and increased rate of recovery. However, they present some drawbacks such as being too expensive and unsuitable for the determination of a large number of molecules. The MycoSep columns are efficient but can only deal with the mixtures of mycotoxins exhibiting similar structures or properties.

Recently, the method based on QuEChERS (quick, easy, cheap, effective, rugged, and safe) has attracted increasing attention in the research field of mycotoxins due to its simplicity and effectiveness for isolating mycotoxins from complex matrices. The successful application of this method has been reported in many products, including cereals and their products [16], spices [17], nuts and seeds [18], Madeira wine [19], human breast milk [20], sesame butter [21], noodles [22], eggs [23], human biological fluids [24], and baby food [25]. In order to reduce the matrix effects (MEs), which can result in unsatisfactory recoveries, additional purification with sorbents such as octadecyl silica (C18), primary secondary amine (PSA), and graphitized carbon black (GCB) are commonly used. The sorbents react differently depending on the physicochemical properties of the compounds constituting the samples [26,27].

The published analyses of multiple mycotoxins in cereals only report the determination of 14 mycotoxins, including deoxynivalenol (DON), aflatoxins (AFs), and fumonisins (FBs) [15,28,29,30,31], but do not take into account enniatins (ENNs) and beauvericin (BEA), which are commonly found in harvested grains [32]. To the best of our knowledge, few studies have been published on the evaluation of the simultaneous presence of 25 mycotoxins in cereals. Although Liu et al. [21] detected 26 mycotoxins in sesame butter, they used a two-ion mode and mobile phase system. For quality and safety risk assessment of cereals and their products, the aim of this study was to develop and validate an efficient and reliable method for the simultaneous quantitative analysis of multiple mycotoxins in cereals. The strategy exploits the efficiency of QuEChERS extractions and the sensitive and selective UPLC–MS/MS technique. We have optimized the mobile phase types, gradients of elution, and the removal of the matrix effects.

## 2. Results and Discussion

### 2.1. Optimization of the Type of the Mobile Phase

The mobile phase plays an important role in the ionization efficiency when the analytes enter the MS/MS system. Acetonitrile, methanol, water, formic acid, and buffers such as ammonium acetate are commonly used as the mobile phase in UPLC. We have selected formic acid and ammonium acetate because of their high ionization efficiency and solubility in the presence of methanol. Therefore, we investigated mixtures of methanol/water and acetonitrile/water, separately, as the mobile phase. The results showed that the response of fumonisins was 15 times higher when using methanol/water than when using acetonitrile/water, with an acceptable peak shape. Moreover, the use of methanol/water produced better separation of the mycotoxins compared with acetonitrile/water. Therefore, we selected methanol as the elution mobile phase.

Adding ammonium acetate (10 mM) and formic acid (0.1%, *v*/*v*) to the mobile phase of methanol improved the responses of all mycotoxins. Adding formic acid greatly enhanced the response especially of positively charged ENNs, BEA, and FBs. Thereby, the formic acid in water was selected to improve the mobile phase. The possible reason for the superior performance is that ionic strength can increase after adding a suitable amount of formic acid solution to the mobile phase; the change in ionic strength can affect the behavior of the separated material, which is advantageous to the positive ion protons.

**Figure 1:**
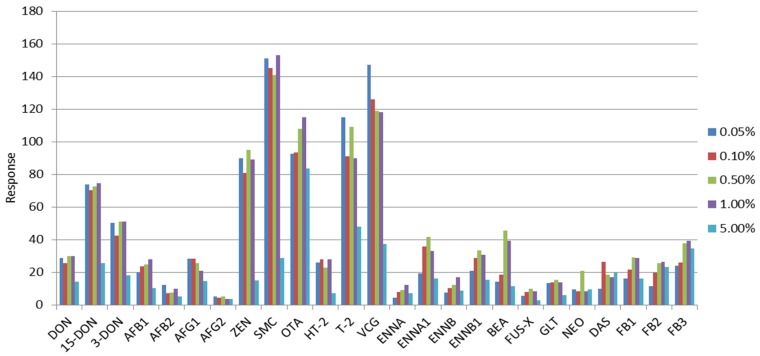
Response obtained for a fixed concentration of the 25 mycotoxins at different concentrations of the formic acid in water. DON: deoxynivalenol; AF: aflatoxin; ZEN: zearalenone; SMC: sterigmatocystin; OTA: ochratoxin; T-2: T-2 toxin; HT-2: HT-2 toxin; VCG: verruculogen; ENN: enniatin; BEA: beauvericin; FUS-X: fusarenon-X; GLT: gliotoxin; NEO: neosolaniol; DAS: 4,5-diacetoxyscirpenol; FB: fumonisin

Varying concentrations of formic acid solution (0.05%, 0.1%, 0.5%, 1.0%, and 5.0%, *v*/*v*) showed minimal effect, but the responses of zearalenone (ZEN), ENN A1, ENN B1, BEA, fusarenon-X (FUS-X), gliotoxin (GLT), and neosolaniol (NEO) were relatively higher with 0.5% formic acid compared to the other proportions (Figure 1). Considering the efficiency of all mycotoxins, 0.5% (*v*/*v*) formic acid in water was selected for the mobile phase.

### 2.2. Optimization of the Gradient Elution

The gradient elution program of mobile phase had effects on the retention time and peak shape of target compounds, and also influenced the ionization efficiency and sensitivity of the target compounds. The gradient elution supports an efficient separation of the multiple mycotoxins, shortens the retention time, and enhances the peak shape and sensitivity.

**Figure 2:**
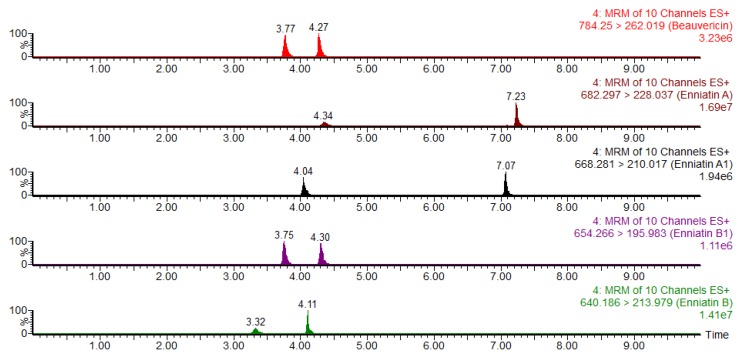
The change of peak shape between ENNs and BEA when using different ratios of formic acid water solution/methanol. MRM: multiple reaction monitoring.

**Figure 3:**
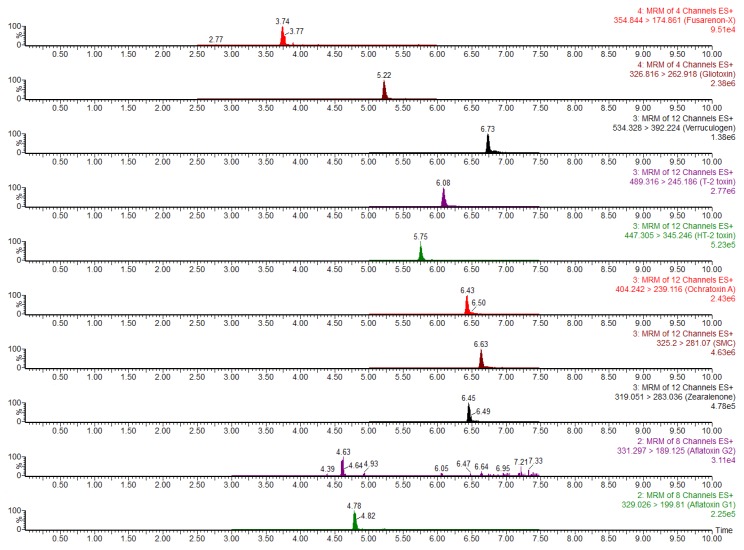

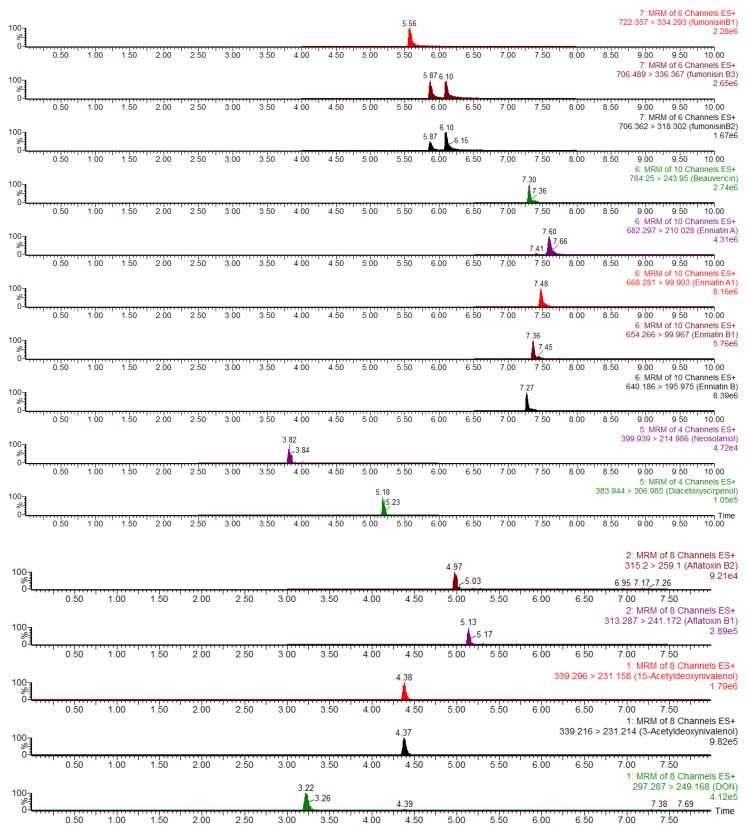
Multiple reaction monitoring (MRM) chromatograms of mixed solutions of 25 mycotoxins standards by positive electrospray ionization (ESI+).

The initial ratio of 0.5% (*v*/*v*) formic acid water solution/methanol was set to 80:20 (*v*/*v*), resulting in a suitable response and peak shape for the ENNs, while there was no response for FBs, ochratoxin A (OTA), DON, 3-AcDON, and 15-AcDON. In this condition, the results showed that when increasing the proportion of methanol, the ENNs and BEA mycotoxins presented two peaks with varying retention times (Figure 2), which can seriously affect the accuracy of qualitative and quantitative results of the compounds. When the initial ratio of 0.5% (*v*/*v*) formic acid water solution/methanol was set to 95:5 (*v*/*v*), we could observe better-resolved peaks. The optimized gradient elution of eluent A (0.5% formic acid water solution) was as follows: starting with 5% and rapidly increasing the proportion to 85% in 5.5 min, slowly increasing to 100% within 5.8 min, then linearly reducing to 5% in 10.0 min. Under this condition, the concentration of DON, 3-AcDON, 15-AcDON, FUS-X, 4,5-diacetoxyscirpenol (DAS), GLT, FB1, FB2, and FB3 is 200 µg·L^−1^; the concentration of ZEN, OTA, verruculogen (VCG), sterigmatocystin (SMC), T-2 toxin (T-2), HT-2 toxin (HT-2), NEO, ENNA, ENNA1, ENNB, ENNB1, and BEA is 50 µg·L^−1^; and the concentrations of AF B1, AF B2, AF G1, and AF G2 are 10, 3, 10, and 3 µg·L^−1^, respectively. The 25 mycotoxins could be monitored as shown in Figure 3. A good peak shape and resolution were achieved for all the target mycotoxins within 10 min except for the conjugations 3-AcDON and 15-AcDON, which were selected as different MS/MS daughter ions for quantification.

### 2.3. Selection of the Extraction Solvent and Purification Evaluation

The extraction solvent was selected according to the method described in a previous work [15]. The extraction yield greatly improved when the acetonitrile/water content gradually increased until reaching the maximum yield at the proportions of 80% and 84%, respectively [33]. In this study, we have made a slight modification. When using pure acetonitrile as the extraction solvent, the yields of ENNs, BEA, and FBs were less than 60% in all matrices. However, when extracting with a solution containing a ratio of 99:1 (*v*/*v*) of 0.1% formic acid water solution/acetonitrile, we observed more prominent intensities of the peaks, especially for the FBs.

Sorbents C18, PSA, GCB, SPE C18, and the amino column (NH_2_) were selected for the purification of mycotoxins. The sorbent C18 can be used to remove the fatty and nonpolar components of the matrix, whereas PSA is mainly used for the removal of organic acids, pigments, and phenol, among others, whereas GCB mainly removes pigments of the crude sample.

We have optimized the purification by separately testing 100 mg of C18, 150 mg of PSA, and 150 mg GCB. The results are summarized in Table 1. The mycotoxins used with PSA and GCB were recovered with less than 50% yield, and the extraction yield with GCB was less than 20%. The low yield can be explained by the high affinity of the mycotoxins to the sorbents, except for DON mycotoxin. Published results show that GCB is suitable for application with biscuits when using an amount smaller than 500 mg with dichloromethane/methanol (80:20, *v*/*v*) [26]. Nevertheless, dichloromethane is toxic and is not recommended. The sorbent GCB was not suitable for the detection of mycotoxins in pomegranate juices [17] and beer-based drinks [2] due to the sorption of the mycotoxins, so we may not choose GCB as an alternative cleaning sorbent.

Using 0.4 g of PSA produced significant improvements in the recovery, reaching yields superior to 80% for DON, AF B1, AF B2, AF G1, AF G2, T-2 toxin, HT-2 toxin, OTA, ZEN, FB1, and FB2 in rice [28], but the yields decreased when using PSA in sesame, with recoveries less than 10% for T-2, HT-2, OTA, ZEN, FB1, FB2, and FB3, and lower than 60% for AF B1. When the amount of PSA increased, the recovery of AF B2 decreased from 70.4% to 36.4%, and did not affect the recoveries of AF G1 and AF G2 [21].

Our results showed that C18 obtained satisfactory yields of about 60.0%~101.7% for most of the mycotoxins, excepted for T-2, HT-2, SMC, NEO, and DAS, which is consistent with the results obtained for sesame butter [21]. However, using a C18 sorbent concentration of 50 mg/mL did not improve the purification of human breast milk [20].

Using a combination of C18 with PSA in the range of 10, 20, 50, and 100 mg showed that when the amount of PSA increased, the recovery of most mycotoxins decreased. However, the mycotoxins VCG, FUS-X, and GLT were not affected, with extraction yields inferior to 30%. Our results demonstrated that the type and quantity of sorbents greatly influence the extraction yield.

Evaluation of the commonly used purification column MycoSep 226, 227, and 400 (Romer Labs Inc., Union, MO, USA) showed that the three columns were all suitable for DON, 3-AcDON, 15-AcDON, T-2, HT-2, VCG, FUS-X, and DAS, with recoveries from 61.3% to 125.1%, with the MycoSep 227 superior to the 226 and 400, although the three MycoSep columns were not suitable for OTA, ENNs, BEA, NEO, and FBs. Our results are consistent with the observations of Sun et al., who reported on the extraction of mycotoxins from wheat, rice, and corn with MycoSep 226 [15], and the observations reported by other researchers extracting other feedstuffs with MycoSep 400 [34].

The comparison of column C18 with amino column NH_2_ showed that FBs and OTA were not properly recovered from the column NH_2_, as reported in the case of multiple mycotoxins determination of sorghum [35].

Satisfactory recoveries were obtained when using column C18 for FBs, compared with a MultiSep 211 FUM purification cartridge [36], with recoveries of 80.9%–97.0%. In addition, the cost of a C18 column is much lower than an immunoaffinity column. The method thus provides a simple and cheap purification. The results showed that the amino column NH_2_ is also suitable for the extraction of ENNs and BEA, although the yields ranged from 61.3% to 83.6%. This work presents the first simple and cost-effective purification of ENNs and BEA.

It can be clearly observed that by optimization of the extraction parameters, satisfactory results could be obtained for most of the mycotoxins, although a few mycotoxins presented low yields. No cleanup was applied for the following experiments of 25 mycotoxins.

### 2.4. Evaluation of the Matrix Effect

**Figure 4:**
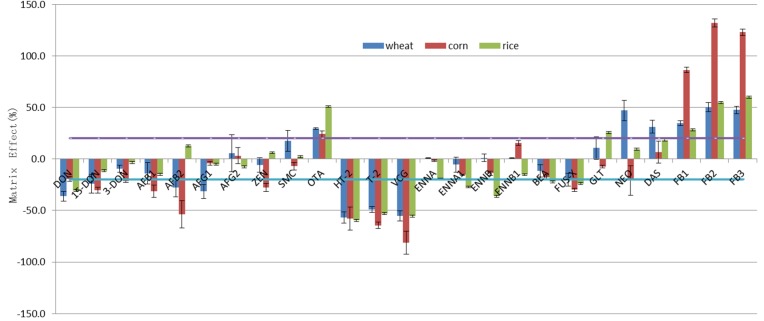
Matrix effects of blank cereals (wheat, corn, and rice) on the response of 25 mycotoxins. The two dashed lines show the tolerance level of the matrix effect.

Matrix effects (MEs) are unavoidable, and the removal of the matrix interference is challenging. At present, reports from scientific literature offer solutions such as the internal standard method using the internal standards zearalenone (ZEN) and deepoxy-deoxynivalenol (DOM) [35] or isotopically labeled standards [37]. Although using a standard curve with an acceptable linear relation presents the advantage of high precision, the choice of an appropriate internal standard for a multicomponent analysis is often difficult and expensive. The response of the target mycotoxins can be suppressed or enhanced on account of the interfering matrix components. The ME was calculated by the Equation (1) for different blanks of wheat, corn, and rice samples as shown in Figure 4.

(1) $$\text{ME}=100\times \left(1-\frac{\text{area of mycotoxin seandard in blank matrix}}{\text{area of mycotoxin standard in solent}}\right)$$

It can be observed that the signal enhancement was prominent for the FBs, with an ME ranging from 28.4% to 132.1%. The effect is emphasized in the case of corn, which exhibited an ME of about twice that of wheat or rice. The signal enhancement of the FBs was also reported in spices (enhancement of about 20%) [38] and sesame butter [21], but the was suppressed in white rice (ME between 13.2% and 17.5%) [9]. The MEs of OTA and DAS were slightly enhanced, presenting values ranging from 24.3% to 50.9% and 6.8% to 31.4%, respectively.

Most of the mycotoxins had a signal suppression effect and were close to the tolerable range between +20% and −20% [23]. However, the signals of HT-2, T-2, and VCG were significantly suppressed by 48.8%–81.3%, as reported for cereal syrups [38].

It is worth mentioning that AF B1 and B2 in corn presented a strong signal suppression effect, as reported in white rice, edible nuts, and seeds by Arroyo-Manzanares et al. [18]. Compared to literature results, we obtained acceptable ME percentages except for AF B1 and B2. The mycotoxins NEO and DAS in wheat showed a strong signal enhancement. The signal suppression for the ENNs, especially for ENNB in rice, was more important than that for wheat and corn.

In conclusion, the matrix effects caused by different matrices were significant for most of the mycotoxins. The calibration curves help to reduce the MEs and, therefore, increase the extraction yields to improve the accuracy of the analysis.

### 2.5. Method Validation

#### 2.5.1. Calibration Curves and Linearity

The calibration curves were constructed using a blank matrix with of the following concentrations: 0.1, 0.2, 0.5, 1, 2, 5, 10, 25, 50, 100, 200, 300, 400, and 500 µg·L^−1^. The peak area (*Y*) was plotted against the concentration of the analyte (*X*). The linear range of DON, 3-AcDON, 15-AcDON, FUS-X, DAS, FB1, FB2, and FB3 was from 0.5 to 500 µg·kg^−1^; the range of ZEN, OTA, VCG, SMC, T-2, HT-2, GLT, NEO, ENNA, ENNA1, ENNB, ENNB1, and BEA was from 0.1 to 200 µg·kg^−1^; and the range for AF B1, AF B2, AF G1, and AF G2 was from 0.1 to 50 µg·kg^−1^ (Table 2). A linear relation was considered to exist when the linear regression coefficient (*r*^2^) was equal to or higher than 0.995.

#### 2.5.2. Limit of Detection and Quantification

Limits of detection (LODs) and limits of quantification (LOQs) were calculated from spiked blank samples at the lowest spiking level. The lowest spiking levels were 3-fold and 10-fold lower than the signal-to-noise ratio (S/N) of the multiple reaction monitoring (MRM) chromatograms. The results showed that LODs were in the range of 0.1–5.0 µg·kg^−1^ except for AF B1, BEA, and ENNs, which were 0.03, 0.05, and 0.05 µg·kg^−1^, respectively, whereas the LOQs were in the range of 0.1–25.0 µg·kg^−1^ (Table 2). These results were far below the values for methanol extraction from cereals and derived products from a study in Tunisia, which reported LODs for ENNA, ENNA1, ENNB, ENNB1, and BEA of 215, 140, 145, 165, and 170 µg·kg^−1^, respectively, and LOQs of 600, 400, 400, 500, and 500 µg·kg^−1^, respectively [39]. The values for FBs were higher than those of the other mycotoxins due to the low signal responses and matrix effects. Nonetheless, when adding the values of FB1 and FB2, we obtain a value which below the maximum limits of European Union regulations (<1000 µg·kg^−1^; FB3 is not included in the regulations). The LODs of regulated mycotoxins (AF B1, AF B2, AF G1, AF G2, DON, ZEN, T-2, HT-2, and OTA) were lower than the maximum limits of the different analytes in regulated cereals per EU regulations [40].

#### 2.5.3. Method Accuracy and Precision

The method accuracy was evaluated by determining the recovery of the standard mycotoxins that were spiked into the blank matrices at three different concentrations as shown in Table 3. The recovery values were in the range of 60.2%–115.8%, and the relative standard deviations (RSDs) of the values were between 0.7% and 19.6%. The results demonstrated that the method applied to the cereals was highly accurate and reliable.

In our study, we also use the certified reference material DON (PriboLab, Level # DW-163) for method verification. The reference material value was 0.5 ± 0.1 µg·kg^−1^, and the actual measured value was 0.44 µg·kg^−1^, which proved the state of instrument is stable and the method is feasible.

The precision of the method was evaluated by intra- and inter-day repeatability and by measuring six times. In the case of the wheat matrices, the intra-day repeatability values were within 2.4%–12.0%, and the inter-day repeatability ranged from 6.1% to 17.6% (Table 2). The results showed that the RSDs of inter-day repeatability test were higher than those for the intra-day repeatability test. Nonetheless, these values were still below 20% and within the allowable range.

### 2.6. Application of the Developed Method on Real Samples

**Figure 5:**
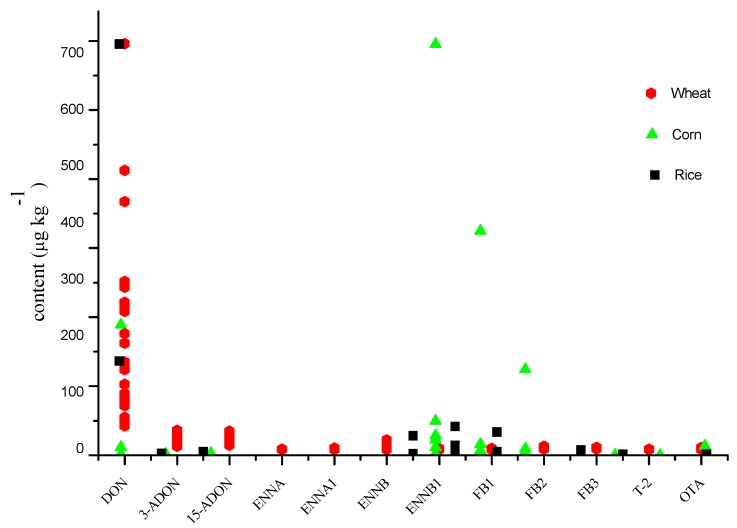
Multiple mycotoxins determination of cereal samples collected from farmland and local supermarkets in China.

The developed and optimized method was applied for the analysis of 65 samples, comprising 26 wheat samples from different areas of China, 14 corn samples collected from two different years (2014 and 2015), and 25 various brands of rice obtained from the local supermarket. As shown in Figure 5, 90% of the samples were contaminated with mycotoxins. The detected mycotoxins were DON, 3-AcDON, 15-AcDON, ENNA, ENNA1, ENNB, ENNB1, FB1, FB2, FB3, T-2, OTA, BEA, ZEN, and SMC. The other mycotoxins (AF B1, AF B2, AF G1, AF G2, HT-2, VCG, NEO, FUS-X, and DAS) were not detected.

In contaminated samples, all the wheat samples contained DON within concentrations ranging from 42.1 to 718.0 µg·kg^−^^1^, but the values are still below the state limit standards. Nevertheless, we should pay attention to the addition of the concentrations of 3-AcDON and 15-AcDON reaching values ranging from 6.1 to 32.8 µg·kg^−1^. It is worth mentioning that 19%, 27%, 69%, and 35% of the total wheat samples were contaminated with ENNA, ENNA1, ENNB, and ENNB1, respectively. The detected concentrations ranged from 0.1 to 17.2 µg·kg^−1^. The concentrations of ENNB were similar to that of ENNA in the wheat sample, as reported in the case of wheat grains from Poland [30] and Morocco [32]. Moreover, the values of FBs (FB1+, FB2+, and FB3), T-2, and OTA ranged from 0.1 to 5.9 µg·kg^−1^, 0.2 to 0.7 µg·kg^−1^, and 0.2 to 3.7 µg·kg^−1^, respectively. These values are all far below the maximum residual limits authorized by the European Union regulations [40].

The main mycotoxins detected in corn (12 samples out of 14 were contaminated) were FBs with concentrations ranging from 0.1 to 1190.7 µg·kg^−1^. Samples from 2014 were more contaminated than the ones from 2015, indicating that storage conditions should be better controlled. The quantity of detected mycotoxins in rice was lower than that of wheat and corn. The concentrations of mycotoxins FBs, OTA, BEA, and SMC ranged from 0.1 to 11.0 µg·kg^−1^, and are below the maximum residual limits, as well as the mycotoxins that are not mentioned in the regulations, such as ENNs and BEA. We should strengthen supervision and try to identify the potential risks and the effect of toxin combinations.

## 3. Conclusions

The parameters of the QuEChERS purification combined with ultrahigh-performance liquid chromatography tandem mass spectrometry were optimized for the simultaneous quantification of 25 mycotoxins in different cereals (wheat, corn, and rice). The method is rapid, simple, and economical. The optimization was focused on the mobile phase type, elution gradient, the extraction solvent, and the matrix effect removal. The method provided a good linear relation, precision, LODs, LOQs, and recoveries. The matrix effect could be controlled by using a matrix-matched calibration. The experimental results also showed that the amino column NH_2_ is suitable for the extraction of ENNs and BEA, whereas the C18 column is suitable for the FBs, which may provide useful suggestion for cleaning up some kinds of mycotoxins in future applications. The optimized method was applied to 26 wheat, 14 corn, and 25 rice samples. We could monitor and examine the potential risk of mycotoxins in cereals by a quick and reliable method.

## 4. Experimental Section

### 4.1. Materials

The reagents methanol, acetonitrile, and formic acid were of HPLC grade and were purchased from Thermo Fisher Scientific Inc. (Shanghai, China). The extraction kits QuEChERS (Part No: 5982-0650, Agilent Technologies Inc., Santa Clara, CA, USA), sorbent C18, GCB, and PSA were purchased from Sigma-Aldrich Corp. (St. Louis, MO, USA). The solid-phase extraction column C18 and the amino column NH_2_ were purchased from Agilent Technologies Inc. (Santa Clara, CA, USA). Ultra-pure water was produced by a Mill-Q system (Millipore, Billerica, MA, USA). All other reagents were of analytical grade.

Solid mycotoxin standards of deoxynivalenol (DON), 3-acetyldeoxynivalenol (3-AcDON), 15-acetyldeoxynivalenol (15-AcDON), fusarenon-X (FUS-X), neosolaniol (NEO), 4,5-diacetoxyscirpenol (DAS), aflatoxin B1 (AF B1), aflatoxin B2 (AF B2), aflatoxin G1 (AF G1), aflatoxin G2 (AF G2), T-2 toxin, HT-2 toxin, fumonisin B1 (FB1), fumonisin B2 (FB2), fumonisin B3 (FB3), sterigmatocystin (SMC), verruculogen (VCG), ochratoxin A (OTA), zearalenone (ZEN), gliotoxin (GLT), enniatin A (ENNA), enniatin A1 (ENNA1), enniatin B (ENNB), enniatin B1 (ENNB1), and beauvericin (BEA) were purchased from Sigma-Aldrich (St. Louis, MO, USA).

### 4.2. Preparation of the Standard Solutions

The standards AF B1, AF B2, AF G1, and AF G2 were dissolved in methanol at concentrations of 200, 60, 200, and 60 µg·L^−1^, respectively. Stock solutions of the other standards were prepared in methanol at a concentration of 100 mg/L. All the standard solutions were stored at −18 °C in darkness. The working standard solutions were freshly prepared before use by diluting the stock solution in order to obtain a 50:50 *v*/*v* ratio of methanol/water. The concentrations of the working mixed solutions were set accordingly to the response values of the mycotoxins by MS. The concentration of the working mixed standard solutions for DON, 3-AcDON, 15-AcDON, FUS-X, DAS, GLT, FB1, FB2, and FB3 were 200 µg·L^−1^. In the case of ZEN, OTA, VCG, SMC, T-2, HT-2, NEO, ENNA, ENNA1, ENNB, ENNB1, and BEA, the concentration of the working mixed solutions was 50 µg·L^−1^. Finally, the concentrations of the working mixed solutions for AF B1, AF B2, AF G1, and AF G2 were 10, 3, 10, and 3 µg·L^−1^, respectively.

A blank substrate was measured with the matrix of a standard solution according to Section 2.3, with the same concentration as the working mixed standard solutions.

### 4.3. Sample Preparation

The sample (2 ± 0.05 g) was finely milled in a 50 mL centrifuge tube before extraction. In the case of spiked sample, the required volume of spiking standard solution was added prior to the extraction step. A mixture of acetonitrile/water (20 mL, 80:20, *v* /*v*) containing 0.1% (*v*/*v*) formic acid was added to the solution. The mixture was then vortexed for 30 s and shaken on an automatic thermostatic cultivation shaker (Yiheng Technology Co., Ltd., Shanghai, China) for 30 min. The QuEChERS extraction kit, containing sodium chloride (1.0 g), anhydrous magnesium sulfate (4.0 g), sodium citrate (1.0 g), and sodium hydrogen citrate sesquihydrate (0.5 g), was added to the mixture and the tube was vigorously shaken by hand for 2 min. The sample was then centrifuged (3-18K, Sigma, St. Louis, MO, USA) at 8000 revolutions per minute (relative centrifugal force, RCF, 3500× *g*) for 5 min at 10 °C. The supernatant (2 mL) containing the extracts was transferred to a centrifuge tube, and submitted to a stream of N_2_ at 55 °C until complete dryness. The residue was sequentially dissolved in a mixture of methanol/water (0.5 mL, 1:1, *v*/*v*), vortexed for 1 min, then filtered through nylon filter (0.22 µm, Membrana GmbH, Wuppertal, Germany), and the extracts were finally recovered in a 200 µL microtube for UPLC–MS/MS analysis.

### 4.4. UPLC–MS/MS Conditions

The extracted mycotoxins were analyzed by ultrahigh-performance liquid chromatography coupled to tandem quadrupole mass spectrometry (UPLC–MS/MS, XEVO-TQ, Waters Corp., Milford, MA, USA). The data processing was performed using the MassLynx 4.0 software (Waters Corp., Milford, MA, USA).

The separation of the mycotoxins was performed using a CORTECS C18 column (100 × 2.1 mm, 1.6 µm, Waters Corp., Manchester, NH, USA) under a flow rate of 200 µL·min^−1^. The volumes of the strong wash (90% methanol) and weak wash (10% methanol) solvents were 100 µL and 600 µL, respectively. The proportion of the mobile phase was set as described in Section 2.2. Mobile phase A was methanol and mobile phase B was a water solution containing 0.5% (*v*/*v*) formic acid. The mobile phase A was gradually eluted from 5% during 5.5 min, 850% during 5.8 min, 100% during 9.0 min, 5% during 10.0 min. The column was maintained at room temperature and the sample temperature was 20 °C. The injection volume was 5 µL.

Tandem mass spectrometry (MS/MS) detection was performed in a positive electrospray ionization mode (ESI+). For the infusion experiments, the mycotoxins standards (0.1 mg/L) were dissolved in methanol and a flow rate of 25 µL·min^−1^ was applied. The parameters were optimized by using the IntelliStart program supplied by Waters Corp. (Milford, MA, USA). The capillary voltage was set at 2.5 kV. Gaseous nitrogen was used as the cone, nebulizing, and desolvation gas. The source and desolvation temperature was 110 °C and 500 °C, respectively. The cone and desolvation gas flows were maintained at 20 L·h^−1^ and 800 L·h^−1^, respectively. The collision gas flow was 0.17 mL·min^−1^. The analysis of the mycotoxins was performed in a multiple reaction monitoring (MRM) mode. For each mycotoxin, at least one precursor ion and two fragment ions were monitored. The most abundant product ion was selected for quantification while the second-most intense ion was used for the qualitative analysis. The shape of the peaks was optimized by waiting 1 min before and after the retention time. The acquisition parameters for the 25 analyzed mycotoxins are summarized in Table 4.

## Figures and Tables

**Table 1:** Recoveries (%) of the 25 mycotoxins using different sorbents for the purification.

Mycotoxin	100 mg C18	100 mg C18 + 10 mg PSA	100 mg C18 + 20 mg PSA	100 mg C18 + 50 mg PSA	100 mg C18 + 100 mg PSA	150 mg PSA	150 mg GCB	MycoSep 226	MycoSep 227	MycoSep 400	SPE C18	SPE NH_2_
DON	82.6	96.5	76.8	85.1	89.0	61.3	58.8	110.0	86.9	87.5	88.2	94.1
15-DON	79.2	81.3	77.1	84.0	78.4	34.9	18.7	125.1	113.8	74.8	78.7	90.3
3-DON	85.3	88.4	68.7	86.9	85.4	30.3	15.8	106.3	100.7	77.4	112.1	112.1
AF B1	67.7	67.9	44.7	17.1	24.2	ND	ND	73.5	ND	68.8	64.6	81.6
AF B2	65.3	62.3	61.0	13.0	15.0	ND	ND	68.7	ND	63.2	71.9	45.0
AF G1	64.7	61.2	64.5	32.7	14.7	3.2	ND	86.1	ND	63.9	67.3	36.4
AF G2	101.7	72.0	103.3	ND	ND	ND	ND	71.6	ND	60.4	61.3	72.1
ZEN	67.0	50.0	18.9	43.8	23.5	22.6	0.7	74.8	0.9	53.9	30.7	49.4
SMC	52.1	38.4	12.7	40.8	39.6	19.6	ND	62.7	ND	36.6	34.0	51.4
OTA	71.8	8.7	23.2	2.6	0.7	21.5	0.5	15.4	4.1	40.1	71.9	5.1
HT-2	36.3	50.1	8.8	42.1	41.5	13.6	8.6	70.6	72.9	67.2	29.2	33.2
T-2	51.9	49.6	14.6	54.0	53.1	22.0	11.2	86.1	94.1	61.7	34.6	37.1
VCG	63.6	67.6	60.4	68.7	85.2	28.1	1.0	69.7	65.2	61.3	26.3	40.2
ENA	82.2	39.1	16.4	34.1	29.3	16.8	4.1	6.1	ND	25.2	6.4	65.0
ENA1	60.0	30.8	15.9	23.3	24.3	26.8	8.1	8.2	ND	28.6	14.4	66.0
ENB	64.4	49.5	47.1	38.0	41.8	35.2	15.2	30.6	ND	39.1	32.1	83.6
ENB1	60.4	33.9	24.8	28.6	30.7	35.4	12.1	17.6	ND	36.1	22.2	74.5
BEA	66.2	33.2	34.6	30.4	47.5	16.2	3.4	35.5	ND	27.2	13.9	61.3
FUS-X	62.2	79.4	83.3	67.6	71.3	19.2	10.3	100.2	94.8	71.1	91.3	77.0
GLT	98.6	82.3	56.3	75.2	66.6	26.7	11.5	21.5	5.0	24.7	69.1	5.5
NEO	28.9	26.0	17.4	27.2	25.2	5.3	13.7	58.4	37.3	42.8	14.0	31.0
DAS	45.9	38.0	49.1	21.8	34.9	31.6	9.8	115.5	108.1	85.1	65.9	51.1
FB1	63.7	3.9	2.5	1.3	0.5	0.2	7.6	2.4	0.8	1.5	117.0	3.5
FB2	89.5	8.2	5.1	3.0	1.4	0.6	4.9	6.4	0.6	4.2	113.3	1.1
FB3	84.5	5.6	4.3	3.0	1.8	0.9	8.5	2.8	0.9	11.7	114.4	3.6

ND: not detected; C18: octadecyl silica; PSA: primary secondary amine; GCB: graphitized carbon black; SPE: solid-phase extraction.

**Table 2:** Fitting equations, linear ranges, correlation coefficients (*r*), limits of detection (LODs), and limits of quantifications (LOQs) of the 25 mycotoxins.

Compound	Calibration Curve	Linear Range	*r*	LOD	LOQ	Intra-Day Precision (*n* = 6)	Inter-Day Precision (*n* = 6)
		(µg·kg^−1^)		(µg·kg^−1^)	(µg·kg^−1^)	RSD (%)	RSD (%)
DON	*Y* = 98.62*X* + 169.00	5.0~500	0.9995	5.0	15.0	3.4	7.0
15-DON	*Y* = 313.95*X* + 1394.48	5.0~500	0.9997	2.0	5.0	3.6	7.1
3-DON	*Y* = 191.02*X* + 375.01	5.0~500	0.9999	2.0	5.0	3.3	7.3
AF B1	*Y* = 853.62*X* + 1079.28	0.1~50	0.9950	0.03	0.1	8.9	7.2
AF B2	*Y* = 1284.87*X* + 470.48	0.3~15	0.9974	0.1	0.3	5.3	9.2
AF G1	*Y* = 945.23*X* + 253.68	0.1~50	0.9999	0.1	0.3	8.1	12.2
AF G2	*Y* = 378.07*X* ± 111.02	0.3~15	0.9960	0.1	0.3	8.8	16.9
ZEN	*Y* = 272.06*X* ± 695.88	1.0~200	0.9993	1.0	4.0	6.4	6.5
SMC	*Y* = 2071.13*X* + 1706.52	0.4~200	0.9998	0.1	0.4	4.2	9.6
OTA	*Y* = 573.77*X* ± 2398.65	0.4~200	0.9997	0.1	0.4	8.4	9.3
HT-2	*Y* = 174.01*X* ± 181.35	0.4~200	0.9996	0.1	0.4	8.3	7.9
T-2	*Y* = 1144.87*X* + 2500.6	0.4~200	0.9996	0.1	0.4	6.1	7.2
VCG	*Y* = 581.24*X* + 1550.22	0.2~200	0.9997	0.1	0.4	8.2	7.4
ENNA	*Y* = 222.75*X* ± 123.09	0.2~200	0.9998	0.05	0.2	7.8	12.6
ENNA1	*Y* = 6333.74*X* + 5213.22	0.2~200	0.9998	0.05	0.2	2.4	11.1
ENNB	*Y* = 6124.97*X* ± 673.19	0.1~200	0.9998	0.05	0.2	5.6	12.9
ENNB1	*Y* = 4212.47*X* + 2487.88	0.1~200	0.9997	0.05	0.2	6.9	14.7
BEA	*Y* = 3170.66*X* + 659.08	0.1~200	0.9999	0.05	0.2	7.3	9.3
FUS-X	*Y* = 28.65*X* ± 15.62	0.5~500	0.9996	10.0	25.0	6.9	9.4
GLT	*Y* = 473.91*X* + 2091.75	5.0~200	0.9993	5.0	10.0	4.6	6.1
NEO	*Y* = 97.93*X* + 1000.67	5.0~200	0.9993	5.0	10.0	7.0	17.6
DAS	*Y* = 97.93*X* + 1000.67	10.0~500	0.9993	10.0	25.0	5.6	14.3
FB1	*Y* = 83.71*X* ± 323.38	25.0~500	0.9992	15.0	25.0	6.3	6.3
FB2	*Y* = 70.66*X* ± 421.58	25.0~500	0.9950	15.0	25.0	12.0	8.5
FB3	*Y* = 60.71*X* + 2650.23	25.0~500	0.9995	15.0	25.0	7.4	7.8

*Y*: peak area, *X*: concentration in µg·L^−1^. RSD: relative standard deviation.

**Table 3:** Recovery and precision of 25 mycotoxins spiked in three different cereal matrices at three concentrations (%RSD of peak areas).

Mycotoxin	Wheat (*n* = 6)			Corn (*n* = 6)			Rice (*n* = 6)		
	Level 1	Level 2	Level 3	Level 1	Level 2	Level 3	Level 1	Level 2	Level 3
DON	115.8 (0.7)	97.9 (5.5)	98.8 (6.8)	98.4 (15.8)	106.6 (5.9)	101.2 (1.9)	97.0 (4.7)	116.9 (5.3)	106.5 (1.0)
15-ADON	91.0 (3.6)	100.8 (4.0)	96.8 (2.6)	88.9 (3.4)	95 (2.5)	97.9 (0.5)	85.8 (3.9)	113.1 (3.4)	93.0 (2.5)
3-ADON	93.4 (2.9)	104.7 (1.4)	96.8 (1.7)	85 (9.7)	93.8 (4.3)	102.5 (4.5)	73.4 (7.2)	100.6 (0.5)	80.9 (2.8)
AF B1	61.7 (10.8)	118.1 (1.7)	87.4 (12.2)	82.3 (9.3)	60.3 (18.5)	90 (5.4)	82.8 (7.0)	91.7 (17.1)	105.0 (5.1)
AF B2	114.0 (18.1)	63.0 (6.2)	71.0 (12.0)	60.6 (11.1)	108.3 (14.9)	91.6 (12.9)	67.9 (6.0)	98.3 (4.7)	79.4 (19.0)
AF G1	84.8 (10.5)	73.2 (12.8)	88.2 (14.8)	73.4 (18.8)	80.7 (8.5)	86.7 (3.4)	88.8 (8.1)	89.4 (19.2)	95.9 (18.6)
AF G2	75.7 (11.4)	91.5 (9.5)	84.5 (5.8)	114.7 (13.4)	89.9 (17.1)	63.7 (19.6)	62.5 (11.8)	103.3 (16.5)	100.4 (8.7)
ZEN	70.0 (6.4)	73.5 (8.5)	63.3 (12.2)	92 (4.4)	68.1 (7.3)	88.6 (5.3)	84.6 (17.1)	64.3 (10.5)	79.2 (2.1)
SMC	64.8 (10.0)	62.8 (5.8)	69.9 (8.4)	69.1 (4.7)	69.1 (3.6)	63.5 (9.1)	66.4 (10.5)	58.2 (1.5)	86.2 (6.2)
OTA	77.4 (1.4)	88.7 (11.2)	60.1 (2.0)	77.8 (7.0)	65.7 (2.7)	65.5 (7.7)	74.0 (8.7)	68.6 (5.9)	81.4 (9.5)
HT-2	77.5 (5.1)	73.0 (11.8)	84.1 (8.3)	73.6 (0.8)	70.8 (10.3)	89.7 (2.8)	83.8 (9.2)	103.6 (4.4)	86.1 (3.3)
T-2	62.4 (8.8)	67.9 (2.5)	69.9 (2.0)	77.0 (5.7)	69.4 (4.0)	85.9 (4.3)	82.2 (3.6)	122.4 (0.6)	85.0 (1.5)
VCG	61.4 (5.5)	106.5 (6.7)	62.4 (6.1)	67.6 (8.4)	71.9 (10.1)	113.2 (0.2)	92.7 (1.4)	79.0 (7.1)	89.5 (9.0)
ENNA	71.2 (9.1)	63.5 (1.5)	67.0 (9.2)	65.7 (6.9)	63.5 (4.0)	89.2 (0.6)	73.9 (5.2)	69.3 (10.6)	99.0 (1.1)
ENNA1	74.5 (3.5)	64.9 (5.3)	61.9 (8.9)	67.2 (4.4)	63.7 (4.9)	75.6 (0.7)	82.3 (11.7)	74.2 (4.4)	73.4 (6.7)
ENNB	67.4 (14.3)	69.7 (5.3)	70.2 (5.1)	71.4 (8.3)	96.6 (13.0)	81.7 (8.7)	104.2 (6.7)	79.3 (2.7)	87.7 (3.9)
ENNB1	67.7 (12.2)	65.0 (11.2)	60.2 (5.0)	64.9 (9.2)	73.1 (1.1)	76.2 (15.4)	80.0 (11.4)	77.3 (2.8)	79.1 (1.0)
BEA	65.4 (7.6)	71.7 (3.3)	60.3 (3.2)	66.5 (3.5)	74.8 (3.9)	65.1 (19.6)	72.8 (9.4)	67.9 (1.6)	62.5 (4.1)
FUS-X	97.5 (14.5)	107.9 (4.9)	119.3 (11.7)	101.4 (4.5)	87.2 (10.)	111.2 (10.6)	76.5 (3.6)	110.4 (19.0)	92.1 (6.9)
GLT	67.7 (1.3)	74.3 (5.2)	82.4 (2.6)	75.7 (3.2)	75.7 (1.0)	79.3 (4.4)	69.9 (3.9)	86.3 (2.5)	74.9 (1.2)
NEO	63.7 (9.5)	69.3 (12.5)	64.6 (1.1)	63.2 (18.5)	95.3 (15.8)	62.6 (15.9)	64.6 (7.6)	108.7 (14.2)	67.2 (10.9)
DAS	77.0 (12.2)	84.0 (14.3)	100.7 (4.1)	70.8 (2.9)	88.1 (1.4)	87.8 (11.7)	78.8 (17.4)	72.6 (4.1)	88.4 (2.5)
FB1	109.4 (4.5)	107.1 (7.8)	73.9 (2.3)	118.9 (2.1)	115.4 (2.8)	83.7 (4.7)	107.8 (3.6)	113.7 (2.8)	80.2 (1.5)
FB2	102.3 (3.2)	102.4 (6.9)	74.7 (2.4)	107.4 (4.9)	110.8 (5.6)	87.8 (2.1)	100.9 (0.7)	99.5 (2.4)	105.7 (2.0)
FB3	115.6 (5.2)	102.8 (5.9)	89.6 (1.9)	115.5 (2.3)	114 (2.7)	89.1 (3.5)	111.2 (5.4)	107.4 (3.7)	91.3 (1.1)

Group 1: DON, 15-AcDON, 3-AcDON, FUS-X, GLT, DAS, FB1, FB2, and FB3 at 100, 200, and 500 µg·kg^−1^; Group 2: ZEN, SMC, OTA, HT-2, T-2, VCG, ENNA, ENNA1, ENNB, ENNB1, BEA, and NEO at 20, 50, and 100 µg·kg^−1^; Group 3: AF B1 and AF G1 at 5, 10, and 25 µg·kg^−1^; Group 4: AF G2 and AF B2 at 1.5, 3.0, and 7.5 µg·kg^−1^.

**Table 4:** Ultrahigh-performance liquid chromatography tandem mass spectrometry (UPLC–MS/MS) acquisition parameters of the 25 mycotoxins.

Mycotoxins	Retention Time (min)	Precursor Ion (*m/z*)	Product Ion (*m/z*)	Dwell Time (s)	Cone Voltage (V)	Collision Energy (V)
DON	3.22	297.28	249.1 (Q)	0.005	20	10
			203.1 (q)	0.005	20	14
15-ADON	4.38	339.3	137.1 (Q)	0.005	22	10
			231.2 (q)	0.005	22	12
3-ADON	4.37	339.2	231.2 (Q)	0.005	20	14
			213.2 (q)	0.005	20	22
AF B1	5.13	313.28	241.1 (Q)	0.005	46	38
			285.0 (q)	0.005	46	22
AF B2	4.97	315.2	259.1 (Q)	0.005	40	30
			287.1 (q)	0.005	40	26
AF G1	4.78	329.03	199.8 (Q)	0.005	40	40
			243.0 (q)	0.005	40	28
AF G2	4.63	331.29	189.1 (Q)	0.005	44	34
			245.1 (q)	0.005	44	28
ZEN	6.45	319.05	283.0 (Q)	0.005	18	22
			187.3 (q)	0.005	18	14
SMC	6.63	325.20	281.0 (Q)	0.005	45	36
			253.1 (q)	0.005	45	42
OTA	6.43	404.24	239.1 (Q)	0.005	26	36
			221.1 (q)	0.005	26	26
HT-2	5.75	447.30	345.2 (Q)	0.005	34	18
			285.1 (q)	0.005	34	22
T-2	6.08	489.31	245.1 (Q)	0.005	42	34
			387.2 (q)	0.005	42	22
VCG	6.73	534.32	392.2 (Q)	0.005	22	18
			191.1 (q)	0.005	22	24
ENNA	7.66	682.29	210.0 (Q)	0.005	48	26
			228.0 (q)	0.005	48	28
ENNA1	7.50	668.28	99.9 (Q)	0.005	48	60
			210.0 (q)	0.005	48	26
ENNB	7.30	640.18	195.9 (Q)	0.005	46	24
			213.9 (q)	0.005	46	26
ENNB1	7.45	654.26	99.9 (Q)	0.005	46	54
			195.9 (q)	0.005	46	24
BEA	7.36	784.24	243.9 (Q)	0.005	48	28
			262.0 (q)	0.005	48	26
FUS-X	3.74	354.84	174.8 (Q)	0.005	18	34
			136.8 (q)	0.005	18	34
GLT	5.22	326.81	262.9 (Q)	0.005	16	10
			244.9 (q)	0.005	16	16
NEO	3.82	399.93	214.9 (Q)	0.005	16	18
			304.9 (q)	0.005	16	14
DAS	5.18	383.94	306.9 (Q)	0.005	18	12
			246.9 (q)	0.005	18	16
FB1	5.56	722.35	334.2 (Q)	0.005	56	42
			352.2 (q)	0.005	56	36
FB2	6.10	706.36	318.3 (Q)	0.005	56	40
			336.3 (q)	0.005	56	38
FB3	5.87	706.48	336.3 (Q)	0.005	44	32
			95.0 (q)	0.005	44	48

Note: 25 mycotoxins standards were dissolved in methanol (0.1 mg/L). The protonated ion peaks (M + 1) (precursor ion, *m*/*z*) of the 25 mycotoxins were determined by full scan under flow injection mode. The fragment ions were obtained on the basis of the MS_2_ (product ion scan) and two fragment ions with relatively higher peak intensities selected as the quantitative and qualitative ion, respectively. Automatic optimization of the mass parameters was conducted with the IntelliStart program from Waters Corp. Q: quantitative ion, q: qualitative ion.
